# Forecasting daily bathtub-drowning mortality in Japan: a comparative analysis of statistical, machine learning, and deep learning approaches

**DOI:** 10.3389/fpubh.2025.1715622

**Published:** 2025-12-10

**Authors:** Yoshiaki Tai, Kenji Obayashi, Yuki Yamagami, Keigo Saeki

**Affiliations:** Department of Epidemiology, Nara Medical University School of Medicine, Nara, Japan

**Keywords:** bath-related deaths, bathtub-drowning, meteorological factors, predictive modeling, machine learning, deep learning, older adults

## Abstract

**Background:**

Japan reports the highest global mortality rate from drowning among older adults, predominantly owing to bathtub-related incidents. Despite sustained public health interventions, this mortality has increased over several decades. Timely warnings advising older adults to avoid unsupervised bathing during high-risk conditions may mitigate this issue; however, no nationwide forecasting model currently exists.

**Methods:**

We integrated death certificate records from 1995–2020 (99,930 bathtub-drowning deaths) with meteorological, temporal, and demographic data across all 47 prefectures (446,359 prefecture-days). Daily mortality counts were modeled using a distributed-lag non-linear model (DLNM), extreme gradient boosting (XGBoost), and long short-term memory network (LSTM). Data were partitioned chronologically into training (1995–2015), validation (2016–2018), and test (2019–2020) sets. Predictive accuracy was evaluated using root mean square error (RMSE) and mean absolute error (MAE), whereas feature importance was quantified via Shapley additive explanations.

**Results:**

During the test period, DLNM, XGBoost, and LSTM exhibited comparable predictive performance (RMSE = 0.577, 0.574, 0.575; MAE = 0.345, 0.333, 0.347, respectively). The most important features across all models were daily mean temperature, prefectural population, and binary prefecture indicators. Restricting DLNM meteorological inputs to routinely forecasted variables—daily maximum and minimum temperatures—did not reduce predictive accuracy [RMSE = 0.577 (95% confidence interval, 0.566–0.590); MAE = 0.344 (95% confidence interval, 0.340–0.349)].

**Conclusions:**

The DLNM-based framework provides a practical means of forecasting the daily bathtub-drowning deaths. Integration into routine meteorological broadcasts and mobile platforms may facilitate timely warnings, prompting older adults to avoid unsupervised bathing on high-risk days, thereby reducing Japan's ongoing preventable bath-related mortality.

## Background

Japan reports the highest global mortality rate from drowning among older adults (1). In 2023, 6,944 drowning deaths were recorded (International Classification of Diseases, 10th revision [ICD-10] code, W65-W74), with 6,333 (91.2%) occurring in bathtubs (ICD-10 code, W65) (2). Among individuals aged ≥ 65, bathtub-drowning deaths (W65) were threefold higher than all traffic-related deaths (ICD-10 code, V01-V98) and represented the leading cause of unintentional external mortality (ICD-10 code, V01-X59) in the 65–79-year-old group (2). When including bath-induced intrinsic events (e.g., myocardial infarction and stroke), annual bath-related deaths are estimated at approximately 20,000 (3). These findings suggest that the supposed health benefits of hot-tub bathing—such as improved sleep (4, 5), mood (6), blood pressure regulation (7), and lower cardiovascular risk (8)—can be offset or surpassed by associated mortality risks in older populations.

Existing interventions aimed at reducing bath-related deaths include public safety advisories (9), which recommend maintaining moderate bathwater temperature, limiting immersion duration, and avoiding alcohol or antihypertensive medication prior to bathing. In addition, recent findings also associate low ambient indoor temperatures with increased thermal stress during bathing (10). However, no empirical evidence currently demonstrates a statistically significant reduction in bath-related mortality attributable to these measures. By contrast, both the absolute number of W65-coded deaths and age-adjusted mortality rate have exhibited a steady upward trajectory over recent decades (11).

Kagoshima Prefecture has recently implemented a novel countermeasure: local television weather forecasts now indicate “high-risk days” for bath-related deaths (12, 13). However, because the underlying prediction model was trained exclusively on Kagoshima-specific data, it is not directly generalizable to other regions. Although a previous study demonstrated an inverse association between outdoor temperature and bathtub-drowning deaths using statistical models (14), it remains unclear whether machine-learning and deep-learning approaches outperform classical statistical models for predicting such deaths. In addition, it is uncertain which predictors—including prefecture-level population size and age structure, and meteorological factors other than temperature—meaningfully contribute to improved predictive performance.

Therefore, we aimed to develop a nationwide predictive model to estimate daily bathtub-drowning deaths per prefecture, based on death certificate records from all regions. We further quantified the relative contribution of each predictor to identify the key drivers of model performance.

## Methods

### Study design

To construct a nationwide forecasting model, we utilized daily counts of bathtub-drowning deaths—classified under ICD-10 code W65 (“accidental drowning and submersion while in a bathtub”)—as the target variable for the study period spanning from 1 January 1995 to 31 December 2020. Predictor variables comprised daily meteorological metrics, temporal indicators, and prefectural attributes, as described in the corresponding subsections. We compared seven modeling techniques: generalized linear models (GLM), generalized additive models (GAM), distributed-lag non-linear models (DLNM), extreme gradient boosting (XGB), random forest (RF), multilayer perceptron (MLP), and long short-term memory (LSTM) networks. Each model estimated (i) the expected daily count of W65-coded deaths and (ii) daily probability of at least one W65-coded death occurring in a given prefecture. The study protocol was approved by the Ethics Committee of Nara Medical University (approval no. 3557).

### Daily count of bathtub-drowning deaths

Under Article 33 of the Statistics Act, we acquired death certificate data from the Ministry of Health, Labor and Welfare spanning from 1 January 1995 to 31 December 2020. The dataset comprises all W65-coded deaths occurring in residential settings. Earlier records were excluded because they relied on ICD-9 coding. For each prefecture, we aggregated the daily count of W65-coded deaths by the residential location of the certificate.

### Meteorological predictors

Meteorological variables—daily mean, maximum, and minimum outdoor temperatures (°C); mean relative humidity (%); mean atmospheric pressure (hPa); and total precipitation (mm)—were sourced from the Japan Meteorological Agency (15). Two temperature-derived indices were computed: day-to-day variation [defined as the difference between the mean temperature of the current and previous day (allowing negative values)] and diurnal range (calculated as the difference between the daily maximum and minimum). Precipitation was recoded to an 11-level ordinal scale (0–10), with 0 indicating no rainfall and 1–10 representing successive deciles of the non-zero distribution. Meteorological records spanning from 11 December 1994 to 31 December 2020 were retained to capture potential lagged exposure effects. For each prefecture, temperature data from its most populated city served as representative values. Finer spatial resolution was not adopted owing to the sparsity of daily bathtub-drowning incidents at the municipal level.

### Temporal structure and specific calendar days

The temporal structure comprised three variables. First, a daily sequential index was constructed, spanning from 1 (1 January 1995) to 9,497 (31 December 2020). Second, the day-of-year variable ranged from 1 to 366; to standardize leap and non-leap years, dates from 1 May onward were incremented by one day in non-leap years, ensuring a consistent 366-day representation. Third, day-of-week was encoded using binary indicators: seven for machine and deep learning models, and six for statistical models, with Friday serving as the reference category. Additionally, three binary calendar indicators were defined: one each for national holidays, New Year's Day, and New Year's Eve. National holidays were defined in accordance with Japan's Act on National Holidays (Act No. 178 of 1984, as amended) (16).

### Population data by prefecture

Annual prefecture-level population counts for 1995–2020 were sourced from the quinquennial Japanese Population Census (17), with the census bureau providing interpolated estimates for the intermediate years. The dataset also includes the proportion of residents aged ≥ 65 years for each prefecture.

### Statistical analysis

Descriptive statistics are reported as means [standard deviations (SDs)], medians [interquartile ranges (IQRs)], and counts [percentages (%)]. Missing meteorological values were imputed by fitting a local cubic spline to the preceding 6 days of observations (t−6 to t−1) at the same site and taking the fitted value at the target date. The predictor set for each comprised meteorological variables—daily mean temperature, diurnal temperature range, day-to-day temperature change, mean relative humidity, mean atmospheric pressure, and precipitation—in conjunction with temporal features (daily sequential index, day-of-year, and day-of-week), calendar indicators (national holidays, New Year's Day, and New Year's Eve), and prefecture-level attributes (binary indicators for each prefecture, population size, and proportion of residents aged ≥ 65 years). Data were partitioned into training (1995–2015), validation (2016–2018), and test (2019–2020) sets to emulate prospective forecasting. Model performance for predicting daily counts of bathtub-drowning deaths was assessed using root mean square error (RMSE), mean absolute error (MAE), and Poisson deviance. For the model predicting the occurrence of daily bathtub-drowning deaths, we calculated the area under the receiver operating characteristic curve (ROC-AUC), the area under the precision-recall curve (PR-AUC), and the Brier score. We obtained 95% confidence intervals (CIs) for each performance metric via 1,000 bootstrap resampling of the test set. Autoregressive models were excluded, as the daily count of bathtub-drowning deaths is unavailable for next-day prediction under the current vital statistics reporting system in Japan.

We employed three complementary regression frameworks—GLM, GAM, and DLNM—to analyse two related outcomes. A binomial model with a logistic link function was used to estimate the occurrence of bathtub-drowning deaths, while a quasi-Poisson model with a log link captured their daily counts. The GLM incorporated only daily maximum and minimum temperatures, serving as a parsimonious baseline. By contrast, the GAM and DLNM utilized the full predictor set. In the GAM, daily mean temperature was modeled as a smooth term to flexibly capture non-linear associations. In the DLNM, delayed and non-linear effects of mean outdoor temperature were represented via a distributed-lag non-linear “cross-basis function.” We modeled the exposure–response with a cubic B-spline with knots at either the 10th, 25th, and 50th, or the 25th, 50th, and 75th temperature percentiles; and the lag–response with a natural cubic spline up to 21 days, using three internal knots on a logarithmic scale (about 1, 3, and 8 days). The long-term trend (sequential day index) and seasonality (day of year) were modeled using natural splines in DLNM, and penalized spline in GAM. An exhaustive grid search compared trend spline basis dimensions of 4, 8, and 12, and seasonal spline basis dimensions of 3, 6, and 9, yielding nine GAM configurations; the DLNM yielded 18 owing to two alternative temperature-knot schemes. In GAMs, these values denote upper bounds on degrees of freedom, with the actual complexity determined via penalisation during model fitting. Model performance was evaluated on the validation set using ROC-AUC for the occurrence outcome and RMSE for the count outcome. The optimal specification was refitted on the combined training and validation sets prior to final testing. GAM and DLNM analyses were conducted in R 4.5 using the mgcv and dlnm packages, respectively (18, 19).

### Machine-learning models

Two XGB decision-tree models were trained using the full predictor set and the train, validation, and test datasets described above. For predicting the daily occurrence of bathtub-drowning deaths (binary outcome), we specified the logistic likelihood and tuned the learning rate (η = 0.01 or 0.10), maximum tree depth (3 or 6), row subsampling fraction (0.70 or 1.00), and column subsampling fraction (0.70 or 1.00). Each candidate model was trained for up to 1,000 boosting iterations, with early stopping triggered after 10 consecutive rounds without validation improvement. The model yielding the highest ROC-AUC on the validation set was selected. For predicting the daily count of bathtub-drowning deaths, we employed a Tweedie distribution with variance-power of 1.5, which accommodates zero inflation and right-skewed counts. The same hyperparameter grid and early-stopping criterion were applied. Model selection was based on minimizing RMSE on the validation set, while the Tweedie negative log-likelihood served as the internal training loss. Finally, for both tasks, the optimal hyperparameter configuration and corresponding number of boosting rounds were retrained on the combined training and validation sets to produce the final models, which were saved for subsequent evaluation on the independent test period. RF analyses are described in the Supplementary Methods. The XGB and RF models were trained in R 4.5 using the packages xgboost and randomForest, respectively (20, 21).

### Deep learning models

MLP and LSTM models were implemented in Python 3.11 with TensorFlow and Optuna (22, 23). Following predictor scaling and one-hot encoding (see Supplementary Methods), data were partitioned chronologically as previously described. Hyperparameter optimisation employed Optuna's tree-structured Parzen estimator (25 trials, seed = 42), exploring architectures with 1–3 hidden layers, 32–128 units per layer, dropout rates of 0.10–0.50, and Adam learning rates from 10^−4^ to 10^−1^ (log-uniform). MLP hidden layers used rectified linear unit activations; LSTM layers retained default tanh activations with sigmoid gating. Each candidate network was trained for up to 50 epochs with a batch size of 32, applying early stopping after five epochs without validation improvement.

For models predicting bathtub-drowning deaths, a single sigmoid unit was employed in the output layer, optimized using binary-cross-entropy loss and selected based on the highest validation ROC-AUC. For models predicting the daily count, the output layer incorporated a single exponential activation to ensure strictly positive outputs, optimized via Poisson deviance and selected by the lowest validation RMSE. The optimal hyperparameter configuration was subsequently retrained on the combined training and validation sets and evaluated on the independent test set.

Within the LSTM architecture, daily meteorological observations were structured into overlapping 21-day sequences. Each sequence of meteorological variables was processed by a two-layer stacked LSTM branch, whose final hidden state was concatenated with static covariates—including temporal indicators, calendar days, and prefectural attributes—and subsequently passed to a dense layer to produce the final prediction.

### SHapley additive exPlanations (SHAP)

We employed SHAP to assess feature importance across the DLNM, XGB, and LSTM models predicting daily bathtub-drowning count (see Supplementary Methods). For each model, the absolute SHAP values were averaged per feature and subsequently aggregated into thematic categories—meteorological variables (daily mean temperature for DLNM; all meteorological inputs for LSTM), prefecture, national holidays, and day-of-week—to generate group-level importance scores for visualization.

### Sensitivity analysis

To improve practical applicability in forecasting daily bathtub-drowning deaths, we re-assessed DLNM, XGB, and LSTM models using a reduced meteorological predictor set limited to daily maximum and minimum temperatures, which are routinely included in public weather forecasts. In addition, because archived temperature forecasts spanning the study period were unavailable, we evaluated the performance of DLNM using emulated forecast data as inputs. We perturbed the observed test-set temperatures by adding stochastic deviations calibrated to the Japan Meteorological Agency's regional, month-specific next-day forecast RMSE (0.9–1.8 °C for daily maxima and minima) (24). As the mean forecast bias is not reported and the variance component cannot be separated from the RMSE, we sampled deviations from normal distributions with means of 0, −1.8, or +1.8 °C and a standard deviation of 1.8 °C (the upper RMSE bound), and formed pseudo-forecasts by adding these deviations to the observations.

To address zero inflation and overdispersion, we refitted the GLM and DLNM with a zero-inflated negative binomial (ZINB) family and trained the LSTM by minimizing the ZINB deviance, replacing the previous quasi-Poisson specification. We fitted the GLM and DLNM using the R package glmmTMB (25), which does not implement GAMs.

To compare performance between the chronological partitioning and a time-series cross-validation scheme, we implemented a rolling (walk-forward) evaluation using DLNM. Each fold comprised an initial 7-year training window, a 2-year validation window, and a 1-year test window, advancing by 1 year at a time (e.g., Fold 1: 1995–2001 train, 2002–2003 validation, 2004 test; Fold 2: 1996–2002 train, 2003–2004 validation, 2005 test; …; Fold 17: 2011–2017 train, 2018–2019 validation, 2020 test). The hyperparameter optimisation and validation procedures were identical to those used for the chronological partitioning. To obtain overall estimates, fold-specific results were aggregated using weights proportional to the number of events in the corresponding test period.

We evaluated small-area calibration using prefecture-level aggregates over the test period. For each prefecture, we summed the observed deaths and expected counts from (i) a DLNM with a fixed model and (ii) one with random prefecture intercepts and prefecture-specific seasonal (day-of-year) random effects (see Supplementary Methods). Calibration-in-the-large used a Poisson model with log expected counts as an offset to estimate a single intercept (values near zero indicate no systematic bias). The calibration slope used a Poisson model relating observed counts to log expected counts with an unconstrained slope (values near one indicate well-scaled contrasts). Inference relied on Wald tests with robust standard errors.

## Results

We analyzed 446,359 prefecture-day records—one per calendar day from 1995 to 2020 across all 47 prefectures—documenting 99,930 residential bathtub-drowning deaths. Missing values for daily maximum temperature (*n* = 25), daily minimum temperature (*n* = 9), relative humidity (*n* = 57), and precipitation (*n* = 5) were imputed by fitting a local cubic spline to the preceding 6 days of observations.

Table 1 presents descriptive statistics for all predictors and the outcome. Although no formal statistical tests were conducted, the summary measures indicate upward temporal trends in daily mean, maximum, and minimum temperatures, and relative humidity. Conversely, prefectural populations have declined, whereas the proportion of residents aged ≥ 65 years has increased. Within the training set, days without bathtub-drowning incidents were more frequent than in the validation and test sets.

**Table 1:** Summary of features and target variables across training, validation, and test datasets.

**Features and target variables**	**Training**	**Validation**	**Test**
	**1995–2015**	**2016–2018**	**2019–2020**
	**(*****n*** = **360,490)**	**(*****n*** = **51,512)**	**(*****n*** = **34,357)**
**Meteorological features**
Daily mean temp, mean (SD), °C	15.5 (8.7)	15.9 (8.8)	16.2 (8.3)
Daily maximum temp, mean (SD), °C	20.0 (8.9)	20.5 (9.0)	20.8 (8.5)
Daily minimum temp, mean (SD), °C	11.7 (8.9)	12.1 (9.0)	12.4 (8.7)
Diurnal temp range, mean (SD), °C	8.3 (3.3)	8.4 (3.2)	8.4 (3.2)
Day-to-day change in mean temp, mean (SD), °C	0.0 (1.9)	0.0 (2.0)	0.0 (1.9)
Relative humidity, mean (SD), %	69.1 (12.5)	70.1 (13.1)	70.8 (13.1)
Precipitation, median (range), mm	0 (0–628)	0 (0–308)	0 (0–401)
Atmospheric pressure, mean (SD), hPa	1,008.0 (11.1)	1,008.4 (11.2)	1,008.4 (11.1)
**Prefectural features**
Population, median (range), thousand persons	1,753 (573–13,515)	1,626 (562–13,887)	1,588 (553–14,048)
Proportion of people ≥ 65 years, mean (SD), %	22.0 (4.4)	29.4 (2.9)	30.5 (3.1)
**Bathtub-drowning deaths**
Yearly number of deaths, median (range)	3,641(3,008–5,606)	5,932 (5,632–6,053)	5,538 (5,410–5,666)
**Frequency of daily deaths per prefecture**
0 deaths, *n* (%)	300,234 (83.3%)	40,224 (78.1%)	26,946 (78.4%)
1 deaths *n* (%)	46,412 (12.9%)	7,988 (15.5%)	5,369 (15.6%)
2 deaths *n* (%)	9,206 (2.6%)	1,997 (3.9%)	1,262 (3.7%)
≥ 3 deaths *n* (%)	4,638 (1.3%)	1,303 (2.5%)	780 (2.3%)

SD, standard deviation; temp, temperature. Daily mean temperature change was calculated as the difference between the mean of the current day and that of the preceding day. n indicates the number of days in each dataset partition (training, validation, and test).

Table 2 summarizes the predictive performance of each model for classifying the occurrence of daily bathtub-drowning deaths. The XGB model performance achieved the highest scores—ROC-AUC 0.804 (95% CI 0.799–0.809), PR-AUC 0.590 (95% CI 0.581–0.599), and Brier score 0.129 (95% CI 0.127–0.131)—though inter-model differences were generally minor, excluding the GLM baseline. Supplementary Tables S1, S2 detail the hyperparameter configurations selected during validation.

**Table 2:** Comparative predictive performance of models in classifying daily bathtub-drowning deaths.

**Predictive models**	**ROC-AUC (95% CI)**	**PR-AUC (95% CI)**	**Brier score (95% CI)**
**Statistical models**
GLM (baseline, parsimonious)	0.678 (0.671–0.685)	0.333 (0.325–0.342)	0.163 (0.160–0.165)
GAM	0.800 (0.795–0.804)	0.583 (0.575–0.592)	0.131 (0.129–0.132)
DLNM	0.800 (0.796–0.805)	0.584 (0.576–0.593)	0.130 (0.128–0.132)
**Machine-learning models**
XGB	0.802 (0.797–0.808)	0.586 (0.575–0.597)	0.129 (0.127–0.131)
RF	0.784 (0.779–0.789)	0.565 (0.555–0.574)	0.136 (0.135–0.138)
**Deep learning models**
MLP	0.800 (0.792–0.807)	0.582 (0.562–0.602)	0.130 (0.127–0.133)
LSTM	0.802 (0.794–0.810)	0.585 (0.565–0.603)	0.129 (0.126–0.132)

Statistical models were formulated to predict a binary outcome, with the DLNM incorporating a 21-day lag structure. The LSTM processed sequential input windows spanning 21 days. DLNM, distributed-lag non-linear model; GAM, generalized additive model; GLM, generalized linear model; LSTM, long short-term memory; MLP, multi-layer perceptron; PR-AUC, precision–recall area under the curve; ROC-AUC, receiver operating-characteristic area under the curve; RF, random forest; XGB, extreme gradient boosting.

**Figure 1:**
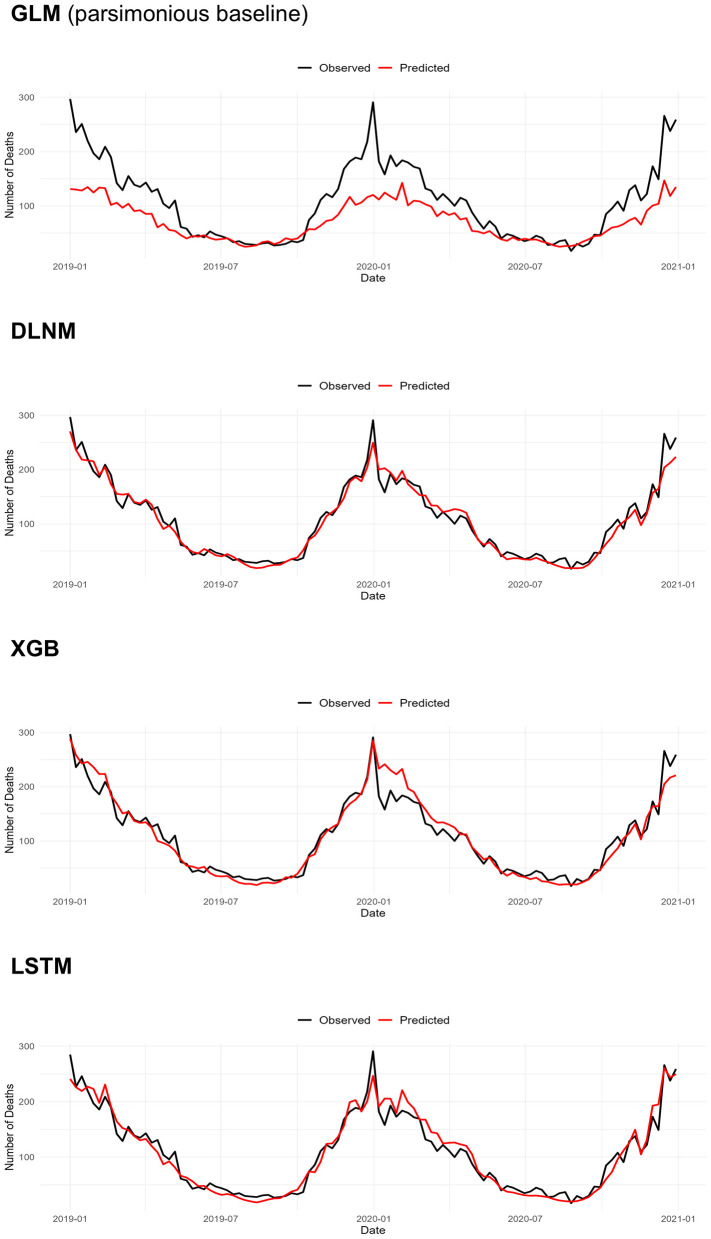
Observed and predicted weekly bathtub-drowning deaths in Japan, test set (2019–2020). Weekly observed and predicted death counts were obtained by summing the corresponding daily values. DLNM, distributed-lag non-linear model; GLM, generalized linear model; LSTM, long short-term memory; XGB, extreme gradient boosting.

Table 3 presents a comparative evaluation of model performance in forecasting daily counts of bathtub-drowning deaths. Overall, the DLNM, XGB, and LSTM models demonstrated comparatively better performance than the remaining models. The XGB model achieved the lowest RMSE [0.574 (95% CI 0.569–0.579)] and MAE [0.333 (95% CI 0.329–0.337)], while DLNM exhibited the lowest Poisson deviance. Predictive accuracy using the reduced meteorological predictor set—limited to daily maximum and minimum temperatures—was comparable to that achieved with the full set (Supplementary Table S3). Moreover, using emulated forecast maxima/minima yielded performance similar to that achieved using the reduced set based on observed temperatures (Supplementary Table S4). Figure 1 displays observed vs. predicted weekly deaths, aggregated from daily counts across all 47 prefectures in the test set; the corresponding daily series is shown in Supplementary Figure S1. GLM underpredicted observed counts in cold months. DLNM predictions closely followed the observed trajectory, including the early weeks of 2020, whereas XGB and LSTM consistently overestimated weekly deaths during this period. Owing to substantial day-to-day variability—even after national aggregation—model differences in Supplementary Figure S1 were less pronounced.

**Table 3:** Comparative predictive performance of models for the daily number of bathtub-drowning deaths.

**Predictive models**	**RMSE (95% CI)**	**MAE (95% CI)**	**Poisson deviance**
**Statistical models**
GLM (baseline, parsimonious)	0.789 (0.763–0.816)	0.421 (0.414–0.428)	24,804.6
GAM	0.583 (0.572–0.595)	0.361 (0.356–0.366)	22,195.4
DLNM	0.577 (0.565–0.590)	0.343 (0.338–0.348)	21,667.7
**Machine-learning models**
XGB	0.574 (0.569–0.579)	0.333 (0.329–0.337)	22,225.5
RF	0.601 (0.596–0.606)	0.426 (0.420–0.432)	24,987.8
**Deep learning models**
MLP	0.586 (0.565–0.604)	0.357 (0.348–0.366)	22,473.0
LSTM	0.575 (0.563–0.586)	0.347 (0.342–0.352)	22,328.1

Statistical models employed a quasi-Poisson distribution, with the DLNM incorporating a 21-day lag structure. The LSTM processed 21-day input windows. DLNM, distributed-lag non-linear model; GAM, generalized additive model; GLM, generalized linear model; LSTM, long short-term memory; MAE, mean absolute error; MLP, multi-layer perceptron; RF, random forest; RMSE, root mean square error; XGB, extreme gradient boosting.

**Figure 2:**
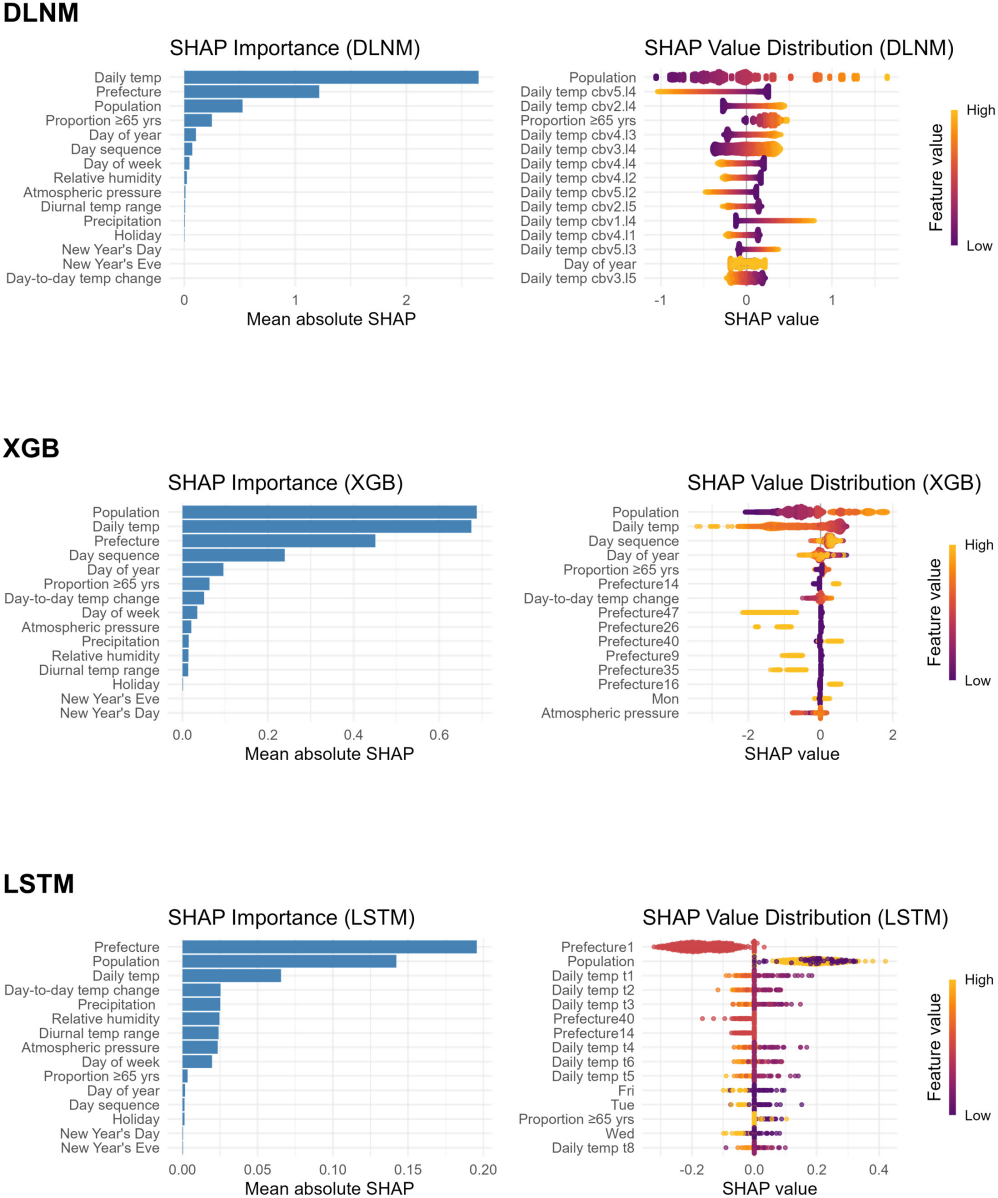
SHAP-based feature importance and impact in predicting the daily number of bathtub-drowning deaths. Mean absolute SHAP values for the predictors “Prefecture,” “Day of week,” “Holiday,” “New Year's Day,” and “New Year's Eve” were computed by aggregating values across their respective one-hot encoded indicators. The mean absolute SHAP value for “Daily temp” was derived by summing contributions from all temperature cross-basis terms (DLNM) or sequence features (LSTM). The SHAP value distribution panel displays only the 15 most influential features. “Daily temp cbv(i).l(j)” denotes the DLNM cross-basis terms combining temperature basis i (i = 1–6) with lag basis j (j = 1–5). “Daily temp (k)” refers to the LSTM input representing the mean temperature recorded k – 1 days prior to the outcome day (k = 1–21). Prefectures 1, 9, 14, 16, 26, 35 40, and 47 correspond to Hokkaido, Tochigi, Kanagawa, Toyama, Kyoto, Yamaguchi, Fukuoka, and Okinawa, respectively. SHAP, SHapley Additive exPlanations; DLNM, distributed-lag non-linear model; XGB, extreme gradient boosting; LSTM, long short-term memory.

Figure 2 presents, for each high-performing model, the aggregated mean absolute SHAP values (bar charts) and corresponding SHAP value distributions for the 15 most influential predictors of daily bathtub-drowning deaths. Across all models, daily mean temperature, prefecture-specific binary indicators, and prefectural population consistently emerged as the most influential features. In particular, higher prefectural population and indicators for high-burden prefectures were associated with increased predicted deaths, whereas higher daily mean temperatures tended to reduce predicted risk. In the SHAP plots, these effects represent each predictor's contribution to deviations from the average forecast. SHAP value distributions from the DLNM and XGB models indicated that higher prefectural populations positively influenced predicted numbers of deaths. Elevated daily mean temperatures were associated with downward shifts in prediction in both XGB and LSTM. By contrast, the DLNM distributes temperature effects across multiple cross-basis terms; therefore, the influence of any individual component cannot be interpreted in isolation. In the LSTM, daily mean temperatures from days immediately preceding the target date exhibited the strongest influence.

Table 4 summarizes the performance of GLM, DLNM, and LSTM under the ZINB specification. The 95% confidence intervals for RMSE and MAE overlapped those obtained using the quasi-Poisson model.

**Table 4:** Comparative predictive performance of statistical models using zero-inflated negative binomial distribution for the daily number of bathtub-drowning deaths.

**Predictive models**	**RMSE (95% CI)**	**MAE (95% CI)**	**Log score (95% CI)**
GLM (baseline, parsimonious)	0.788 (0.763–0.815)	0.420 (0.413–0.427)	0.705 (0.693–0.718)
DLNM	0.592 (0.578–0.606)	0.340 (0.335–0.345)	0.579 (0.570–0.589)
LSTM	0.574 (0.559–0.592)	0.352 (0.343–0.361)	0.566 (0.553–0.580)

The DLNM incorporated a 21-day lag. CI, confidence interval; DLNM, distributed-lag non-linear model; GLM, generalized linear model; LSTM, long short-term memory; MAE, mean absolute error; RMSE, root mean square error.

DLNM performance across 17 walk-forward time-series cross-validation folds is summarized in Supplementary Table S5. Aggregating across folds using death counts as weights, the RMSE and MAE were 0.549 and 0.322, respectively. For folds 16 and 17 (test years 2019 and 2020), which were comparable to those from the time-holdout split. The observed-to-predicted ratio of the DLNM results is shown in Supplementary Table S6. Calibration-in-the-large (intercept) was 0.047 (SE 0.0095; Wald z = 4.80; *p* < 0.001) in the fixed-effects model and −0.135 (SE 0.0095; Wald z = −14.15; *p* < 0.001) in the random-effects model. The calibration slope was 1.012 (SE 0.0095; test of slope = 1: z = 1.24; *p* = 0.214) and 0.984 (SE 0.0113; test of slope = 1: z = −1.40; p = 0.163) in the fixed- and random-effects models, respectively, showing that deviation from unity was not significant.

## Discussion

Leveraging 26 years of death-certificate data linked with meteorological, temporal, and prefectural data across all 47 Japanese prefectures, we developed nationwide predictive models for both the daily incidence and occurrence of bathtub-drowning deaths at the prefectural level. The DLNM, XGB, and LSTM models outperformed the other algorithms, albeit with marginal gains. Moreover, XGB and LSTM did not outperform the DLNM, a classical statistical approach, possibly because DLNM already incorporates flexible non-linear and lagged relationships for temperature and calendar structure. In these three models, daily mean temperature, prefecture-specific binary indicators, and population size were the most influential predictors, a pattern that is consistent with the data structure: temperature primarily captures short-term temporal fluctuations in the risk of bathtub-drowning deaths, whereas prefecture-level indicators and population size capture baseline differences in risk between prefectures. Notably, models constrained to the two routinely broadcast variables—daily maximum and minimum temperatures—achieved comparable accuracy to those utilizing the full meteorological feature set, highlighting their practical applicability. This pattern held when forecast inputs were emulated by adding calibrated noise to the maxima and minima, yielding accuracy broadly comparable to that of the full-feature models.

This study is the first to formally develop and validate nationwide predictive models for bath-related drowning integrating advanced algorithms—including DLNM, XGB, and LSTM—with daily mortality data and a comprehensive set of meteorological, temporal, demographic, and prefecture-level features. Prior studies have predominantly focused on specific regions and employed limited meteorological variables or exploratory modeling techniques (12, 13, 26, 27). Kagoshima's weather broadcast, for instance, identifies high-risk days using a basic linear regression model based solely on local temperature (13), despite its outcome data being rigorously verified by forensic experts. However, this approach lacks generalisability beyond the prefecture. Indeed, our results showed that the parsimonious baseline GLM without temporal and prefectural features underperformed. Similarly, the web-based “Heat-Shock Forecast” (Japan Weather Association and Tokyo Gas) issues alerts based on the indoor–outdoor temperature differential (28, 29), yet its outcome data, predictor variables, and modeling methodology remain undisclosed. Moreover, even within other nationwide datasets (30), household temperature measurements are scarce in certain prefectures and likely exhibit substantial variability due to differences in housing conditions.

The modest performance gains observed for DLNM, XGB, and LSTM relative to baseline models may be attributed to random variability stemming from the low daily incidence of bathtub-drowning deaths. Although leveraging 26 years of nationwide data stabilized expected daily counts, the stochastic nature of the observed deaths constrained both discrimination and calibration, as illustrated in Supplementary Figure S1. This limitation was most pronounced in the classification task, where any count ≥ 1 was collapsed to a single event, discarding information that may vary with the predictors. Future enhancements will require aggregating data across prefectures to increase the number of bathing-related incidents—including drowning deaths and fatal or non-fatal cases of myocardial infarction, arrhythmia, stroke, hyperthermia, and relevant conditions—thereby reducing random error and enhancing signal strength.

Variations in feature importance across DLNM, XGB, and LSTM models likely arise from their distinct mechanisms for encoding temperature effects and handling high-dimensional input data. DLNM employed a cross-basis representation of daily mean temperature with lags up to 21 days, yielding a non-linear exposure–lag surface that dominated its importance profile. The LSTM processed all meteorological variables as 21-day sequences; nonetheless, the aggregated contribution of temperature lags exceeds that of the other weather-related inputs. In contrast, prefecture-level covariates exhibited greater importance in both XGB and LSTM, potentially due to the greedy split-selection inherent in tree-based and gradient-optimized models (31, 32), wherein binary prefecture indicators and population size rapidly minimize the loss function and are thus prioritized during early training.

The findings support the development of a DLNM–based forecasting system to estimate daily bathtub-drowning mortality risk and to disseminate timely alerts via routine weather broadcasts and mobile platforms. Given that DLNM achieved predictive performance comparable to XGB and LSTM while maintaining transparency and ease of implementation, it represents the most pragmatic option for operational integration. Including next-day risk levels for bathtub-drowning deaths alongside routine regional weather forecasts on television and radio could help older people avoid unsupervised bathing. It may also enable family members and carers to encourage safer bathing practices. In future implementations, mobile applications could incorporate personalized risk factors such as age, sex, alcohol consumption, and medical history, and provide push notifications advising older users to avoid unsupervised hot-tub bathing on high-risk days.

This study benefits from the utilization of nationwide mortality data related to bathtub-drowning and the integration of a comprehensive set of meteorological, temporal, and demographic predictors assessed across multiple modeling algorithms. We additionally benchmarked performance under alternative distributional assumptions, evaluated out-of-sample generalisability using both a time-holdout split and walk-forward cross-validation, and examined regional heterogeneity by comparing fixed-effects and random-effects specifications. However, several limitations warrant consideration. First, the outcome was restricted to W65-coded deaths, thereby excluding bath-associated deaths due to intrinsic medical causes, such as myocardial infarction and stroke. As death certificates rarely specify whether such intrinsic deaths occurred in the bathroom, a dedicated surveillance infrastructure would be required for systematic capture. Second, low daily case counts at the prefecture level limited predictive resolution across all models, as previously discussed. Third, we trained the models on observed meteorological inputs rather than forecast data. Nevertheless, when evaluated using emulated forecast temperatures, performance was broadly comparable to that obtained based on observed inputs. Fourth, long-term trends were modeled using a sequential day index, which may not extrapolate reliably over extended horizons; periodic model recalibration is therefore advised. Finally, analyses were conducted at the prefecture rather than municipal level. While finer spatial resolution could enhance local applicability, the increased random error associated with rarer events may negate any gain in accuracy.

## Conclusions

We developed and evaluated predictive models to estimate daily prefecture-level risk of bathtub-drowning mortality across Japan. The DLNM demonstrated superior predictive accuracy under both the comprehensive covariate set and a reduced specification limited to meteorological variables. Based on these findings, integrating DLNM-based forecasts into routine weather forecasts and digital alert systems may help support safer bathing behavior and could contribute to reducing bath-related deaths.

## Data Availability

The data analyzed in this study is subject to the following licenses/restrictions: data from individual vital statistics used in this study cannot be shared, as the authors do not have the right to distribute this information. However, the deidentified public data utilized in this study, along with a data dictionary, are publicly available and can be accessed directly without request. Requests to access these datasets should be directed to https://www.mhlw.go.jp/stf/toukei/goriyou/chousahyo.html.
