# Supplementary material for: Forecasting daily bathtub-drowning mortality in Japan: a comparative analysis of statistical, machine learning, and deep learning approaches
Source: Front Public Health. 2025 Dec 10;13:1715622. doi: 10.3389/fpubh.2025.1715622 (PMC12727947; doi:10.3389/fpubh.2025.1715622)
Supplement: Supplementary file 1 [file Data_Sheet_1.docx]

**Supplementary Methods**

***Random-forest model***

Random‑forest (RF) models were trained on the full predictor set, which comprised daily meteorological metrics (daily mean temperature, diurnal temperature range, day‑to‑day temperature change, mean relative humidity, mean atmospheric pressure, and precipitation); temporal indicators (daily sequential index, day‑of‑year, day‑of‑week, national holidays, and New Year’s Day/Eve); and prefecture attributes (binary indicators for each prefecture, prefectural population, and the proportion of residents aged ≥ 65 years). Data were partitioned chronologically into a training period (1995–2015), a validation period (2016–2018), and a test period (2019–2020).

An exhaustive grid search was conducted on the training set to tune three hyper‑parameters simultaneously: the number of trees (300, 500, 700, or 1000), the number of variables randomly sampled at each split (0.5, 1.0, or 1.5 times the square root of the total predictor count, $\sqrt{p}$), and the minimum number of observations allowed in terminal nodes (1, 5, or 10). For the binary outcome representing the daily occurrence of bathtub‑drowning deaths, candidate models were ranked by the area under the receiver‑operating‑characteristic curve (ROC-AUC) on the validation set, and the configuration with the highest ROC-AUC was selected. For the count outcome representing the daily number of deaths, each hyper‑parameter combination was evaluated on the validation set using root‑mean‑square error (RMSE) and mean absolute error (MAE); the specification yielding the lowest RMSE—500 trees, $\sqrt{p}$ variables per split, and a terminal‑node minimum of one observation—was chosen.

The optimal models were then refitted on the combined training and validation data and finally assessed once on the untouched 2019–2020 test set to estimate out‑of‑sample performance. All analyses were performed in R 4.5 with the randomForest package.^1^

***Standardisation and Normalisation of features for Deep-learning models***

All preprocessing was conducted in Python with pandas.^2^ Continuous meteorological variables—daily mean temperature, day‑to‑day temperature change, diurnal temperature range, relative humidity, and mean atmospheric pressure—together with the log‑transformed prefectural population and the proportion of residents aged ≥ 65 years were z‑score standardised. The sequential time index, day‑of‑year ordinal, and ordinal scale of precipitation were rescaled to the [0, 1] interval using min–max normalisation. Categorical predictors (day of week, prefecture, national holidays, New Year’s Day, and New Year’s Eve) were converted to binary indicator columns via one‑hot encoding. The resulting standardised, normalised, and encoded features were concatenated to create the final predictor matrix for the deep‑learning models.

***Calculations of SHapley Additive exPlanations (SHAP) values***

SHAP values for the distributed lag non-linear model (DLNM) were derived analytically. Each test‑set design vector was centered on the training‑set mean, multiplied element‑wise by the corresponding regression coefficients, and offset by the model intercept; spline bases representing the long‑term trend and the annual cycle were aggregated to generate composite “Time‑trend” and “Season” effects.

For the XGB model, the training design matrix was reconstructed for every test observation, with any columns absent from the test data padded with zeros. XGB’s exact contribution algorithm then produced the bias term and individual‑feature SHAP values. Basis functions that captured the same construct (for example, trend or season) were collapsed, and feature identifiers were mapped to descriptive labels.

Feature importance for the long short-term memory (LSTM) network was assessed with Kernel SHAP. The trained model was reloaded and its two inputs—a 21‑day sequence of meteorological variables and a vector of static covariates (temporal indices, calendar indicators, and prefecture variables)—were concatenated into a single flattened array so that each element could be treated as an independent feature. Fifty randomly selected test observations served as the background distribution, and SHAP values were estimated for 400 additional test cases using 100 Monte Carlo samples per case. The procedure returned a vector of expected predictions and a matrix of SHAP contributions whose rows summed to each model output; these results, together with the corresponding feature matrix and a JSON file of feature names, were stored in compressed NumPy format for visualisation in R with shapviz.^3,4^

***Distributed lag non-linear model with prefecture random intercepts and prefecture-specific seasonal (day-of-year) random effects***

We represented seasonality by day-of-year in two layers. First, we estimated a single smooth, cyclic seasonal curve for Japan as a whole. Secondly, we added a prefecture-specific random seasonal effect: each prefecture was allowed its own smooth deviation from the national curve, treated as a random effect with partial pooling. This approach lets the timing and amplitude of seasonal peaks vary by prefecture while shrinking estimates toward zero where information is limited, thereby reducing overfitting. The smoothness (i.e., resistance to abrupt changes) and the overall degree of pooling were estimated from the data by restricted maximum likelihood. We also included a prefecture random intercept to capture time-invariant baseline differences.

1. Breiman L. Random Forests. *Machine Learning* 2001; **45**(1): 5–32.

2. The pandas development team. pandas-dev/pandas: Pandas (v2.3.1). Zenodo; 2025.

3. Mayer M. shapviz: SHAP visualizations. Comprehensive R Archive Network; 2025.

4. Harris CR, Millman KJ, van der Walt SJ, et al. Array programming with NumPy. *Nature* 2020; **585**(7825): 357–62.

**Supplementary Figure**

**Figure S1**. Observed and predicted daily bathtub-drowning deaths in Japan, test set (2019–2020).


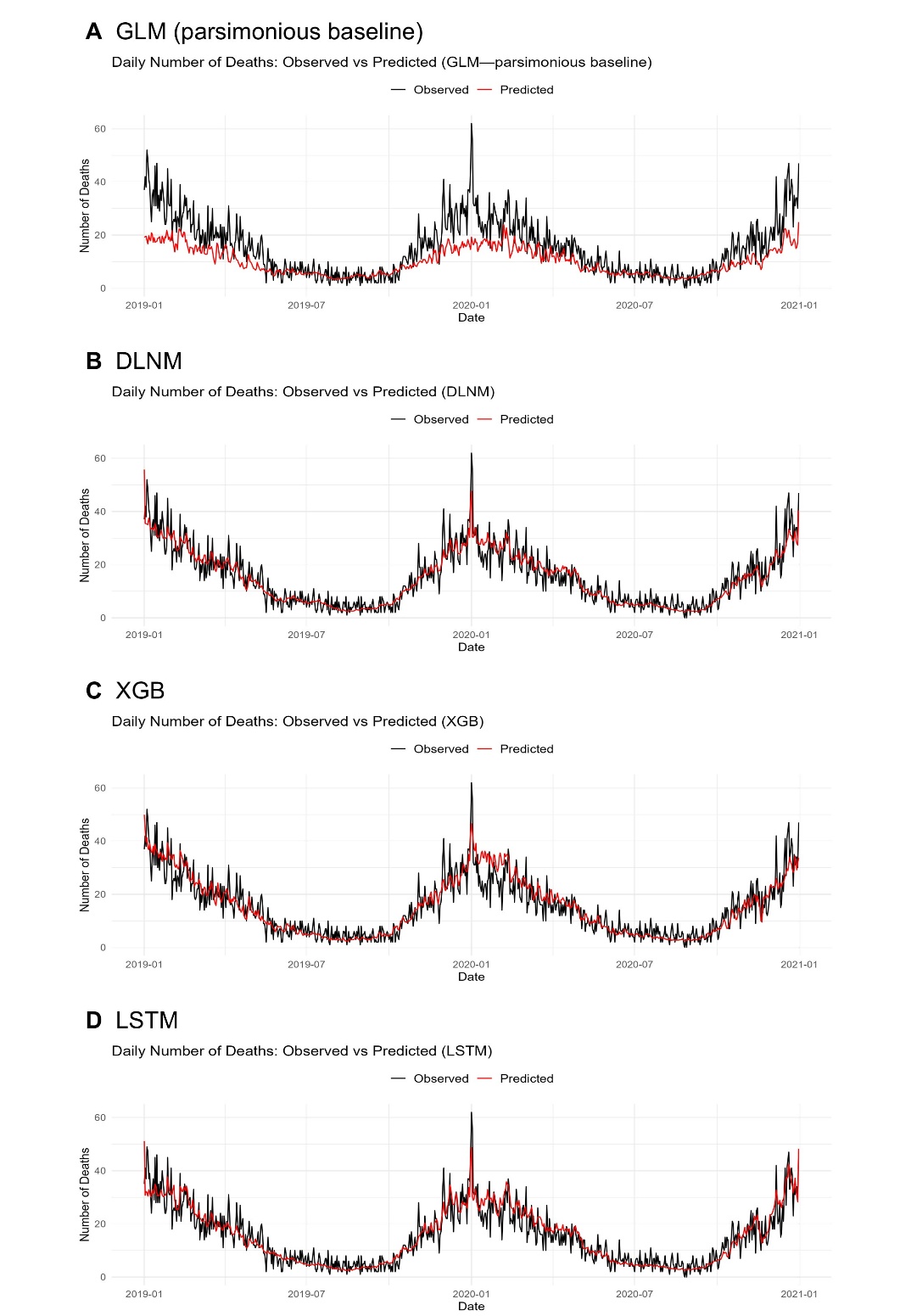


DLNM, distributed-lag non-linear model; GLM, generalised linear model; LSTM, long short-term memory; XGB, extreme gradient boosting.

**Supplementary Tables**

**Table S1**. Lists of the hyperparameters selected during validation for statistical models

| Hyperparameters | GLM | |  | GAM | |  | DLNM | |
| --- | --- | --- | --- | --- | --- | --- | --- | --- |
|  | Binary | Count |  | Binary | Count |  | Binary | Count |
|  |  |  |  |  |  |  |  |  |
| Trend spline df (day sequence) | 12 | 12 |  | 3 | 3 |  | 8 | 12 |
| Seasonal spline df (day-of-year) | 9 | 9 |  | 8 | 2 |  | 6 | 9 |
| Knot locations (temperature percentiles) | — | — |  | — | — |  | 25th/50th/75th | 25th/50th/75th |

df, degrees of freedom; DLNM, distributed-lag non-linear model; GAM, generalised additive model; GLM, generalised linear model.

**Table S2**. Lists of the hyperparameters selected during validation for machine- and deep-learning models

| Hyperparameters | XGB | |  | RF | |  | MLP | |  | LSTM | |
| --- | --- | --- | --- | --- | --- | --- | --- | --- | --- | --- | --- |
|  | Binary | Count |  | Binary | Count |  | Binary | Count |  | Binary | Count |
|  |  |  |  |  |  |  |  |  |  |  |  |
| Learning rate | 0.1 | 0.1 |  | — | — |  | — | — |  | — | — |
| Maximum tree depth | 3 | 6 |  | — | — |  | — | — |  | — | — |
| Row subsampling fraction | 1 | 1 |  | — | — |  | — | — |  | — | — |
| Column subsampling fraction | 1 | 1 |  | — | — |  | — | — |  | — | — |
|  |  |  |  |  |  |  |  |  |  |  |  |
| Number of trees | — | — |  | 500 | 700 |  | — | — |  | — | — |
| mtry | — | — |  | 8 | 4 |  | — | — |  | — | — |
| Node size | — | — |  | 1 | 5 |  | — | — |  | — | — |
|  |  |  |  |  |  |  |  |  |  |  |  |
| Number of hidden layers | — | — |  | — | — |  | 2 | 2 |  | 3 | 2 |
| Units per layer | — | — |  | — | — |  | 64 | 64 |  | 64 | 128 |
| Dropout rate | — | — |  | — | — |  | 0.224 | 0.118 |  | 0.222 | 0.385 |
| Learning rate | — | — |  | — | — |  | 0.0011 | 0.0004 |  | 0.00046 | 0.00037 |

LSTM, long short-term memory; MLP, multi-layer perception; RF, random forest; XGB, extreme gradient boosting.

**Table S3**. Comparative predictive performance of models using daily maximum/minimum temperatures for the daily number of bathtub-drowning deaths

|  | RMSE (95% CI) | MAE (95% CI) | Poisson deviance |
| --- | --- | --- | --- |
|  |  |  |  |
| DLNM | 0.577 (0.566–0.590) | 0.344 (0.340–0.349) | 21793.5 |
| XGB | 0.584 (0.572–0.596) | 0.345 (0.340–0.350) | 22356.4 |
| LSTM | 0.577 (0.565–0.589) | 0.348 (0.343–0.353) | 22389.7 |
|  |  |  |  |

A DLNM assumed a quasi-Poisson distribution, incorporating a 21-day lag. XGB was trained with Tweedie loss. Deep-learning models minimised Poisson negative log-likelihood with a soft-plus output; the LSTM ingested 21-day input windows.

DLNM, distributed-lag non-linear model; LSTM, long short-term memory; XGB, extreme gradient boosting.

**Table S4**. Comparing DLNM predictions using emulated forecasts of daily maximum and minimum temperatures for the daily number of bathtub-drowning deaths

| μ (bias) | σ (SD) | RMSE (95% CI) | MAE (95% CI) | Poisson deviance |
| --- | --- | --- | --- | --- |
| 0 | 1.8 | 0.578 (0.566–0.591) | 0.344 (0.340–0.349) | 21802.4 |
| +1.8 | 1.8 | 0.578 (0.567–0.591) | 0.344 (0.340–0.349) | 21806.0 |
| −1.8 | 1.8 | 0.578 (0.566–0.591) | 0.344 (0.340–0.349) | 21799.0 |

μ (bias) and σ (SD) denote the mean and standard deviation of the additive random noise injected into the observed temperature inputs of the test datasets (independent across days).

CI: confidence interval; DLNM: distributed-lag non-linear model; MAE: mean absolute error; RMSE: root mean square error; SD: standard deviation.

**Table S5**. DLNM performance with walk-forward validation

| Fold | Train window | Validation window | Test year | RMSE | MAE | Trend spline df (day sequence) | Seasonal spline df (day-of-year) |
| --- | --- | --- | --- | --- | --- | --- | --- |
| 1 | 1995–2001 | 2002–2003 | 2004 | 0.436 | 0.246 | 4 | 9 |
| 2 | 1996–2002 | 2003–2004 | 2005 | 0.481 | 0.265 | 12 | 3 |
| 3 | 1997–2003 | 2004–2005 | 2006 | 0.470 | 0.275 | 4 | 6 |
| 4 | 1998–2004 | 2005–2006 | 2007 | 0.465 | 0.261 | 8 | 9 |
| 5 | 1999–2005 | 2006–2007 | 2008 | 0.497 | 0.289 | 8 | 3 |
| 6 | 2000–2006 | 2007–2008 | 2009 | 0.503 | 0.291 | 4 | 3 |
| 7 | 2001–2007 | 2008–2009 | 2010 | 0.518 | 0.299 | 8 | 9 |
| 8 | 2002–2008 | 2009–2010 | 2011 | 0.567 | 0.322 | 4 | 3 |
| 9 | 2003–2009 | 2010–2011 | 2012 | 0.588 | 0.346 | 8 | 9 |
| 10 | 2004–2010 | 2011–2012 | 2013 | 0.573 | 0.345 | 8 | 6 |
| 11 | 2005–2011 | 2012–2013 | 2014 | 0.575 | 0.326 | 8 | 3 |
| 12 | 2006–2012 | 2013–2014 | 2015 | 0.572 | 0.339 | 8 | 9 |
| 13 | 2007–2013 | 2014–2015 | 2016 | 0.577 | 0.340 | 4 | 6 |
| 14 | 2008–2014 | 2015–2016 | 2017 | 0.615 | 0.364 | 8 | 3 |
| 15 | 2009–2015 | 2016–2017 | 2018 | 0.593 | 0.357 | 4 | 9 |
| 16 | 2010–2016 | 2017–2018 | 2019 | 0.579 | 0.346 | 4 | 9 |
| 17 | 2011–2017 | 2018–2019 | 2020 | 0.565 | 0.346 | 8 | 3 |

df: degrees of freedom; DLNM: distributed-lag non-linear model; MAE: mean absolute error; RMSE: root mean square error.

**Table S6**. Observed-to-expected ratios from fixed-effects and random-effects DLNMs for bathtub-drowning deaths

| Prefecture | Observed (O), n | Fixed-effects model | |  | Random-effects model * | |
| --- | --- | --- | --- | --- | --- | --- |
|  |  | Expected (E), n | O/E |  | Expected (E), n | O/E |
| 1 | 486 | 287.75 | 1.69 |  | 545.40 | 0.89 |
| 2 | 161 | 80.87 | 1.99 |  | 123.43 | 1.30 |
| 3 | 84 | 96.14 | 0.87 |  | 125.76 | 0.67 |
| 4 | 176 | 175.71 | 1.00 |  | 216.17 | 0.81 |
| 5 | 173 | 162.63 | 1.06 |  | 103.87 | 1.67 |
| 6 | 138 | 157.31 | 0.88 |  | 111.12 | 1.24 |
| 7 | 172 | 143.35 | 1.20 |  | 180.27 | 0.95 |
| 8 | 174 | 196.24 | 0.89 |  | 260.59 | 0.67 |
| 9 | 57 | 75.29 | 0.76 |  | 179.71 | 0.32 |
| 10 | 221 | 233.69 | 0.95 |  | 184.51 | 1.20 |
| 11 | 193 | 202.56 | 0.95 |  | 656.16 | 0.29 |
| 12 | 127 | 215.69 | 0.59 |  | 579.02 | 0.22 |
| 13 | 525 | 615.30 | 0.85 |  | 1235.92 | 0.42 |
| 14 | 1953 | 1683.44 | 1.16 |  | 860.80 | 2.27 |
| 15 | 246 | 329.65 | 0.75 |  | 237.26 | 1.04 |
| 16 | 232 | 210.46 | 1.10 |  | 102.00 | 2.27 |
| 17 | 126 | 110.47 | 1.14 |  | 110.62 | 1.14 |
| 18 | 97 | 122.63 | 0.79 |  | 72.26 | 1.34 |
| 19 | 129 | 95.66 | 1.35 |  | 85.93 | 1.50 |
| 20 | 353 | 261.15 | 1.35 |  | 265.04 | 1.33 |
| 21 | 283 | 179.33 | 1.58 |  | 193.20 | 1.46 |
| 22 | 296 | 292.73 | 1.01 |  | 344.21 | 0.86 |
| 23 | 634 | 580.29 | 1.09 |  | 738.21 | 0.86 |
| 24 | 119 | 174.15 | 0.68 |  | 184.00 | 0.65 |
| 25 | 147 | 121.71 | 1.21 |  | 145.47 | 1.01 |
| 26 | 39 | 55.09 | 0.71 |  | 267.28 | 0.15 |
| 27 | 673 | 750.30 | 0.90 |  | 954.75 | 0.70 |
| 28 | 589 | 581.56 | 1.01 |  | 596.84 | 0.99 |
| 29 | 54 | 79.93 | 0.68 |  | 148.13 | 0.36 |
| 30 | 101 | 98.41 | 1.03 |  | 99.78 | 1.01 |
| 31 | 54 | 51.87 | 1.04 |  | 59.72 | 0.90 |
| 32 | 51 | 49.64 | 1.03 |  | 79.08 | 0.64 |
| 33 | 111 | 147.46 | 0.75 |  | 201.67 | 0.55 |
| 34 | 143 | 205.05 | 0.70 |  | 317.83 | 0.45 |
| 35 | 44 | 56.64 | 0.78 |  | 155.14 | 0.28 |
| 36 | 66 | 59.67 | 1.11 |  | 83.23 | 0.79 |
| 37 | 41 | 58.50 | 0.70 |  | 106.39 | 0.39 |
| 38 | 112 | 93.05 | 1.20 |  | 158.95 | 0.70 |
| 39 | 48 | 55.76 | 0.86 |  | 78.10 | 0.61 |
| 40 | 879 | 828.34 | 1.06 |  | 579.63 | 1.52 |
| 41 | 78 | 73.19 | 1.07 |  | 88.18 | 0.88 |
| 42 | 179 | 128.65 | 1.39 |  | 157.07 | 1.14 |
| 43 | 157 | 102.57 | 1.53 |  | 197.52 | 0.79 |
| 44 | 132 | 87.57 | 1.51 |  | 137.39 | 0.96 |
| 45 | 104 | 84.23 | 1.23 |  | 117.13 | 0.89 |
| 46 | 114 | 111.51 | 1.02 |  | 171.23 | 0.67 |
| 47 | 5 | 9.53 | 0.52 |  | 74.53 | 0.07 |

Observed and model-predicted death counts were aggregated over the test period (2019–2020).

* Includes prefecture random intercepts and prefecture-specific seasonal (day-of-year) random effects.

DLNM: distributed-lag non-linear model.
